# 2-Ethyl 4-methyl 5-ethyl-3-methyl-1*H*-pyrrole-2,4-dicarboxyl­ate

**DOI:** 10.1107/S1600536812001729

**Published:** 2012-01-21

**Authors:** Gui-Fen Lu, Min Zhu, Wei-Hua Zhu, Zhong-Ping Ou

**Affiliations:** a School of Chemistry and Chemical Engineering, Jiangsu University, Zhenjiang 212013, People’s Republic of China

## Abstract

The title pyrrole derivative compound, C_12_H_17_NO_4_, was synthesized from methyl 3-oxopenta­noate by a Knorr-type reaction and contains a pyrrole ring to which two diagonal alk­oxy­carbonyl groups and two diagonal alkyl substituents are attached. The methyl­carbonyl and ethyl­carbonyl substituents are approximately co-planar with the pyrrole ring, making dihedral angles of 5.64 (2) and 3.44 (1)°, respectively. In the crystal, adjacent mol­ecules are assembled by pairs of N—H⋯O hydrogen bonds into dimers in a head-to-head mode.

## Related literature

For applications of polysubstituted pyrroles, see: Brockmann & Tour, (1995 ▶); Guilard *et al.* (2001 ▶); Trofimov *et al.* (2004 ▶). For related structures, see: Lu *et al.* (2011 ▶); Takaya *et al.* (2001 ▶). For complexes of pyrrole derivatives, see: Fan *et al.* (2008 ▶); Ou *et al.* (2009 ▶); Paixão *et al.* (2003 ▶); Yamamoto *et al.* (1986 ▶).
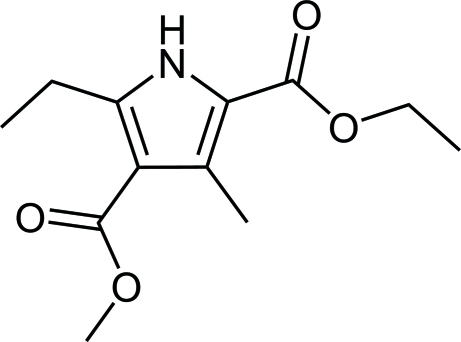


## Experimental

### 

#### Crystal data

C_12_H_17_NO_4_
*M* *_r_* = 239.27
Triclinic, 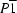

*a* = 7.2827 (10) Å
*b* = 8.8573 (12) Å
*c* = 11.1806 (16) Å
α = 77.948 (2)°
β = 73.135 (2)°
γ = 69.970 (2)°
*V* = 643.62 (15) Å^3^
*Z* = 2
Mo *K*α radiation
μ = 0.09 mm^−1^
*T* = 293 K
0.15 × 0.12 × 0.06 mm

#### Data collection

Bruker APEXII CCD area-detector diffractometer
Absorption correction: multi-scan (*SADABS*; Sheldrick, 2003 ▶) *T*_min_ = 0.986, *T*_max_ = 0.995
3249 measured reflections
2255 independent reflections
1891 reflections with *I* > 2σ(*I*)
*R*_int_ = 0.018

#### Refinement

*R*[*F* ^2^ > 2σ(*F* ^2^)] = 0.067
*wR*(*F* ^2^) = 0.220
*S* = 1.11
2255 reflections
159 parameters
1 restraint
H atoms treated by a mixture of independent and constrained refinement
Δρ_max_ = 0.51 e Å^−3^
Δρ_min_ = −0.36 e Å^−3^

### 

Data collection: *APEX2* (Bruker, 2004 ▶); cell refinement: *SAINT-Plus* (Bruker, 2001 ▶); data reduction: *SAINT-Plus*; program(s) used to solve structure: *SHELXS97* (Sheldrick, 2008 ▶); program(s) used to refine structure: *SHELXL97* (Sheldrick, 2008 ▶); molecular graphics: *XP* in *SHELXTL* (Sheldrick, 2008 ▶); software used to prepare material for publication: *SHELXL97*.

## Supplementary Material

Crystal structure: contains datablock(s) global, I. DOI: 10.1107/S1600536812001729/vm2149sup1.cif

Structure factors: contains datablock(s) I. DOI: 10.1107/S1600536812001729/vm2149Isup2.hkl

Supplementary material file. DOI: 10.1107/S1600536812001729/vm2149Isup3.cml

Additional supplementary materials:  crystallographic information; 3D view; checkCIF report

## Figures and Tables

**Table 1:** Hydrogen-bond geometry (Å, °)

*D*—H⋯*A*	*D*—H	H⋯*A*	*D*⋯*A*	*D*—H⋯*A*
N1—H1*A*⋯O1^i^	0.84 (1)	2.07 (1)	2.883 (3)	165 (2)

Symmetry code: (i) 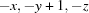.

**Fig. 1.**
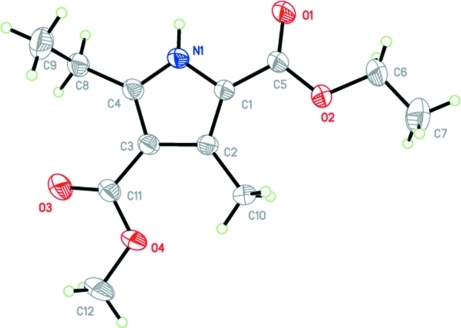
Molecular structure with the unique atom-labelling scheme. Displacement ellipsoids are drawn at the 30% probability level.

**Fig. 2.**
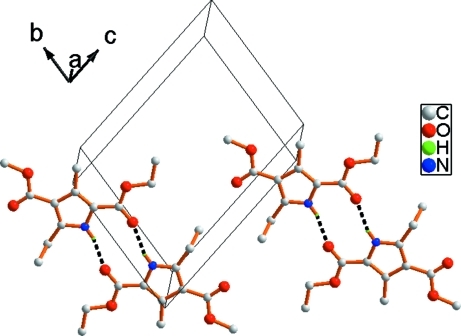
Dimer formation in the crystal packing.

## supplementary crystallographic information

### Comment

Polysubstituted pyrroles have been paid much attention because of their wide application in the preparation of porphyrins (Trofimov *et al.*, 2004), corroles (Guilard *et al.*, 2001) and as monomers for polymer chemistry (Brockmann & Tour, 1995; Paixão *et al.*, 2003). In view of the importance of the 2-(alkoxycarbonyl)pyrrole derivatives (Fan *et al.*, 2008; Lu *et al.*, 2011; Takaya *et al.*, 2001), the title compound was synthesized and characterized by X-ray diffraction.

As shown in Fig. 1, the compound has a five-membered pyrrole ring as skeleton and four substituents. The methoxycarbonyl and ethoxycarbonyl groups are located on two diagonal carbon atoms of the pyrrole skeleton, which is also true for the methyl and ethyl substituents, forming an asymmetrical molecule. Adjacent molecules are assembled in a head to head mode by hydrogen bonding between the donor atom N_1_ and acceptor atom O_1_ (symmetry code: -*x*, 1 - *y*, -*z*) (Table 1, Fig. 2). The bond distances are in the normal range of the similar species reported by Yamamoto *et al.* (1986).

### Experimental

The title compound was synthesized from ethyl acetoacetate and methyl 3-oxopentanoate through oximination, Claisen condensation and reductive condensation according to the method reported by Ou *et al.* (2009). Single crystals suitable for X-ray measurements were grown from ethanol by slowly evaporation at room temperature.

### Refinement

All the non-hydrogen atoms were refined anisotropically by full-matrix least-squares calculations on F^2^. All H atoms (except H1a) were placed in geometrically idealized positions and treated as riding on their parent atoms with C—H = 0.97 Å, *U*_iso_ = 1.2U_eq_ (C) for methylene atoms and C—H = 0.96 Å, *U*_iso_ = 1.5U_eq_ (C) for methyl atoms. The H1a atom has located in a difference map and refined with *U*_iso_ = 1.5U_eq_ (N). The command '*DFIX*' has been used to restrain the distance of H1a—N1 = 0.83 Å.

### Crystal data

**Table:**

C_12_H_17_NO_4_	*Z* = 2
*M**_r_* = 239.27	*F*(000) = 256
Triclinic, *P*1	*D*_x_ = 1.235 Mg m^−^^3^
Hall symbol: -P 1	Mo *K*α radiation, λ = 0.71073 Å
*a* = 7.2827 (10) Å	Cell parameters from 1663 reflections
*b* = 8.8573 (12) Å	θ = 2.4–26.8°
*c* = 11.1806 (16) Å	µ = 0.09 mm^−^^1^
α = 77.948 (2)°	*T* = 293 K
β = 73.135 (2)°	Sheet, colorless
γ = 69.970 (2)°	0.15 × 0.12 × 0.06 mm
*V* = 643.62 (15) Å^3^	

### Data collection

**Table:**

Bruker APEXII CCD area-detector diffractometer	2255 independent reflections
Radiation source: fine-focus sealed tube	1891 reflections with *I* > 2σ(*I*)
graphite	*R*_int_ = 0.018
φ and ω scans	θ_max_ = 25.0°, θ_min_ = 2.9°
Absorption correction: multi-scan (*SADABS*; Sheldrick, 2003)	*h* = −8→6
*T*_min_ = 0.986, *T*_max_ = 0.995	*k* = −10→9
3249 measured reflections	*l* = −13→13

### Refinement

**Table:**

Refinement on *F*^2^	Secondary atom site location: difference Fourier map
Least-squares matrix: full	Hydrogen site location: inferred from neighbouring sites
*R*[*F*^2^ > 2σ(*F*^2^)] = 0.067	H atoms treated by a mixture of independent and constrained refinement
*wR*(*F*^2^) = 0.220	*w* = 1/[σ^2^(*F*_o_^2^) + (0.1366*P*)^2^ + 0.1514*P*] where *P* = (*F*_o_^2^ + 2*F*_c_^2^)/3
*S* = 1.11	(Δ/σ)_max_ < 0.001
2255 reflections	Δρ_max_ = 0.51 e Å^−^^3^
159 parameters	Δρ_min_ = −0.36 e Å^−^^3^
1 restraint	Extinction correction: *SHELXL97* (Sheldrick, 2008), Fc^*^=kFc[1+0.001xFc^2^λ^3^/sin(2θ)]^-1/4^
Primary atom site location: structure-invariant direct methods	Extinction coefficient: 0.046 (17)

### Special details

**Table:**

Geometry. All e.s.d.'s (except the e.s.d. in the dihedral angle between two l.s. planes) are estimated using the full covariance matrix. The cell e.s.d.'s are taken into account individually in the estimation of e.s.d.'s in distances, angles and torsion angles; correlations between e.s.d.'s in cell parameters are only used when they are defined by crystal symmetry. An approximate (isotropic) treatment of cell e.s.d.'s is used for estimating e.s.d.'s involving l.s. planes.
Refinement. Refinement of *F*^2^ against ALL reflections. The weighted *R*-factor *wR* and goodness of fit *S* are based on *F*^2^, conventional *R*-factors *R* are based on *F*, with *F* set to zero for negative *F*^2^. The threshold expression of *F*^2^ > σ(*F*^2^) is used only for calculating *R*-factors(gt) *etc*. and is not relevant to the choice of reflections for refinement. *R*-factors based on *F*^2^ are statistically about twice as large as those based on *F*, and *R*- factors based on ALL data will be even larger.

### Fractional atomic coordinates and isotropic or equivalent isotropic displacement parameters (Å^2^)

**Table:**

	*x*	*y*	*z*	*U*_iso_*/*U*_eq_	
C10	0.3898 (4)	−0.0962 (3)	−0.1197 (2)	0.0550 (6)	
H10A	0.4158	−0.2064	−0.0808	0.082*	
H10B	0.5149	−0.0720	−0.1553	0.082*	
H10C	0.3217	−0.0814	−0.1849	0.082*	
C11	0.2132 (4)	−0.1884 (3)	0.1807 (2)	0.0560 (6)	
C12	0.3494 (6)	−0.4707 (4)	0.1703 (3)	0.0963 (11)	
H12A	0.4249	−0.5445	0.1089	0.144*	
H12B	0.2222	−0.4899	0.2098	0.144*	
H12C	0.4232	−0.4871	0.2328	0.144*	
O4	0.3164 (3)	−0.3073 (2)	0.10932 (18)	0.0785 (6)	
O3	0.1512 (4)	−0.2162 (3)	0.29184 (19)	0.0950 (8)	
H1A	0.020 (3)	0.3365 (14)	0.079 (2)	0.052 (7)*	
C1	0.1913 (3)	0.1820 (3)	−0.04297 (19)	0.0461 (5)	
C2	0.2603 (3)	0.0150 (2)	−0.02273 (19)	0.0439 (5)	
C3	0.1853 (3)	−0.0280 (3)	0.1078 (2)	0.0470 (6)	
C4	0.0707 (3)	0.1150 (3)	0.1615 (2)	0.0491 (6)	
C5	0.2114 (4)	0.3014 (3)	−0.1527 (2)	0.0569 (6)	
C6	0.3253 (8)	0.3461 (4)	−0.3746 (3)	0.1147 (15)	
H6A	0.1902	0.4037	−0.3852	0.138*	
H6B	0.3889	0.4250	−0.3721	0.138*	
C7	0.4382 (8)	0.2543 (6)	−0.4772 (3)	0.1304 (17)	
H7A	0.4460	0.3259	−0.5547	0.196*	
H7B	0.3730	0.1779	−0.4801	0.196*	
H7C	0.5715	0.1974	−0.4659	0.196*	
C8	−0.0423 (4)	0.1442 (3)	0.2940 (2)	0.0621 (7)	
H8A	−0.1565	0.2409	0.2921	0.075*	
H8B	−0.0940	0.0540	0.3344	0.075*	
C9	0.0830 (5)	0.1639 (4)	0.3707 (3)	0.0857 (9)	
H9A	0.0028	0.1823	0.4542	0.129*	
H9B	0.1322	0.2546	0.3324	0.129*	
H9C	0.1946	0.0676	0.3748	0.129*	
N1	0.0782 (3)	0.2382 (2)	0.07013 (17)	0.0503 (5)	
O1	0.1384 (3)	0.4462 (2)	−0.15035 (18)	0.0821 (7)	
O2	0.3170 (3)	0.2368 (2)	−0.25857 (16)	0.0748 (6)	

### Atomic displacement parameters (Å^2^)

**Table:**

	*U*^11^	*U*^22^	*U*^33^	*U*^12^	*U*^13^	*U*^23^
C10	0.0654 (14)	0.0407 (12)	0.0528 (13)	−0.0105 (10)	−0.0084 (10)	−0.0104 (10)
C11	0.0628 (13)	0.0460 (13)	0.0535 (13)	−0.0180 (10)	−0.0088 (10)	0.0016 (10)
C12	0.130 (3)	0.0388 (15)	0.097 (2)	−0.0178 (16)	−0.016 (2)	0.0119 (14)
O4	0.1114 (15)	0.0334 (10)	0.0680 (12)	−0.0114 (9)	−0.0062 (11)	0.0025 (8)
O3	0.1393 (19)	0.0586 (12)	0.0578 (12)	−0.0263 (12)	0.0044 (12)	0.0097 (9)
C1	0.0501 (11)	0.0386 (11)	0.0423 (11)	−0.0088 (8)	−0.0071 (8)	−0.0033 (8)
C2	0.0436 (10)	0.0389 (11)	0.0467 (11)	−0.0113 (9)	−0.0087 (8)	−0.0047 (8)
C3	0.0480 (11)	0.0404 (11)	0.0495 (12)	−0.0137 (9)	−0.0083 (9)	−0.0025 (9)
C4	0.0478 (11)	0.0438 (12)	0.0483 (12)	−0.0118 (9)	−0.0047 (9)	−0.0027 (9)
C5	0.0677 (14)	0.0397 (12)	0.0484 (13)	−0.0075 (10)	−0.0055 (10)	−0.0003 (9)
C6	0.183 (4)	0.0601 (18)	0.0511 (17)	−0.014 (2)	0.007 (2)	0.0112 (14)
C7	0.186 (4)	0.123 (3)	0.0515 (19)	−0.033 (3)	−0.008 (2)	0.001 (2)
C8	0.0667 (14)	0.0542 (14)	0.0494 (13)	−0.0144 (11)	0.0032 (11)	−0.0029 (10)
C9	0.104 (2)	0.101 (2)	0.0500 (15)	−0.0386 (19)	−0.0048 (14)	−0.0093 (15)
N1	0.0533 (10)	0.0370 (10)	0.0479 (11)	−0.0050 (8)	−0.0044 (8)	−0.0040 (8)
O1	0.1135 (15)	0.0386 (10)	0.0600 (11)	−0.0033 (9)	0.0023 (10)	0.0007 (8)
O2	0.1088 (14)	0.0453 (10)	0.0434 (10)	−0.0087 (9)	0.0013 (9)	−0.0001 (7)

### Geometric parameters (Å, °)

**Table:**

C10—C2	1.500 (3)	C4—C8	1.498 (3)
C10—H10A	0.9600	C5—O1	1.211 (3)
C10—H10B	0.9600	C5—O2	1.331 (3)
C10—H10C	0.9600	C6—C7	1.428 (5)
C11—O3	1.197 (3)	C6—O2	1.447 (3)
C11—O4	1.330 (3)	C6—H6A	0.9700
C11—C3	1.463 (3)	C6—H6B	0.9700
C12—O4	1.436 (3)	C7—H7A	0.9600
C12—H12A	0.9600	C7—H7B	0.9600
C12—H12B	0.9600	C7—H7C	0.9600
C12—H12C	0.9600	C8—C9	1.491 (4)
C1—N1	1.380 (3)	C8—H8A	0.9700
C1—C2	1.381 (3)	C8—H8B	0.9700
C1—C5	1.451 (3)	C9—H9A	0.9600
C2—C3	1.422 (3)	C9—H9B	0.9600
C3—C4	1.401 (3)	C9—H9C	0.9600
C4—N1	1.335 (3)	N1—H1A	0.839 (10)
			
C2—C10—H10A	109.5	O2—C5—C1	113.5 (2)
C2—C10—H10B	109.5	C7—C6—O2	108.8 (3)
H10A—C10—H10B	109.5	C7—C6—H6A	109.9
C2—C10—H10C	109.5	O2—C6—H6A	109.9
H10A—C10—H10C	109.5	C7—C6—H6B	109.9
H10B—C10—H10C	109.5	O2—C6—H6B	109.9
O3—C11—O4	121.4 (2)	H6A—C6—H6B	108.3
O3—C11—C3	126.0 (2)	C6—C7—H7A	109.5
O4—C11—C3	112.6 (2)	C6—C7—H7B	109.5
O4—C12—H12A	109.5	H7A—C7—H7B	109.5
O4—C12—H12B	109.5	C6—C7—H7C	109.5
H12A—C12—H12B	109.5	H7A—C7—H7C	109.5
O4—C12—H12C	109.5	H7B—C7—H7C	109.5
H12A—C12—H12C	109.5	C9—C8—C4	113.3 (2)
H12B—C12—H12C	109.5	C9—C8—H8A	108.9
C11—O4—C12	117.6 (2)	C4—C8—H8A	108.9
N1—C1—C2	108.30 (19)	C9—C8—H8B	108.9
N1—C1—C5	117.4 (2)	C4—C8—H8B	108.9
C2—C1—C5	134.3 (2)	H8A—C8—H8B	107.7
C1—C2—C3	105.90 (18)	C8—C9—H9A	109.5
C1—C2—C10	126.4 (2)	C8—C9—H9B	109.5
C3—C2—C10	127.7 (2)	H9A—C9—H9B	109.5
C4—C3—C2	107.89 (19)	C8—C9—H9C	109.5
C4—C3—C11	122.8 (2)	H9A—C9—H9C	109.5
C2—C3—C11	129.3 (2)	H9B—C9—H9C	109.5
N1—C4—C3	107.36 (19)	C4—N1—C1	110.53 (19)
N1—C4—C8	121.0 (2)	C4—N1—H1A	125.5 (17)
C3—C4—C8	131.6 (2)	C1—N1—H1A	124.0 (17)
O1—C5—O2	122.4 (2)	C5—O2—C6	117.1 (2)
O1—C5—C1	124.1 (2)		

### Hydrogen-bond geometry (Å, °)

**Table:**

*D*—H···*A*	*D*—H	H···*A*	*D*···*A*	*D*—H···*A*
N1—H1A···O1^i^	0.84 (1)	2.07 (1)	2.883 (3)	165 (2)

Symmetry codes: (i) −*x*, −*y*+1, −*z*.
